# Term neonatal admissions for hypoglycaemia in England and Wales, 2012–2020: a population-based study using the National Neonatal Research Database

**DOI:** 10.1136/archdischild-2025-328872

**Published:** 2025-12-03

**Authors:** Behrouz Nezafat Maldonado, Frances Conti-Ramsden, Tim J van Hasselt, Jessica Fleminger, Lucy C Chappell, Cheryl Battersby, on behalf of the UK Neonatal Collaborative

**Affiliations:** 1 School of Public Health, Imperial College London, London, UK; 2 Centre for Paediatrics and Child Health, Imperial College London, London, UK; 3 King’s College London, London, UK; 4 School of Healthcare, University of Leicester, Leicester, UK; 5 Department of Women and Children’s Health, King’s College London, London, UK

**Keywords:** Neonatology, Intensive Care Units, Neonatal, Health services research

## Abstract

**Objective:**

To evaluate the impact of a change in national guidance on the management of hypoglycaemia on UK term neonatal unit (NNU) admissions, describe perinatal risk factors for hypoglycaemia and identify opportunities to reduce term infant hypoglycaemia admissions.

**Design:**

Retrospective observational cohort study, the UK National Neonatal Research Database.

**Patients:**

Term infant NNU admissions in England and Wales, 2012–2020.

**Main outcome measures:**

Term admission rate to NNU primarily for hypoglycaemia/1000 term live births. Change between epoch 1 (36 months before the publication of the British Association of Perinatal Medicine (BAPM) Framework in April 2017) and epoch 2 (36 months after the publication of the BAPM Framework in April 2017).

**Results:**

Term admissions primarily for hypoglycaemia decreased from 4.9 (95% CI 4.8 to 5.1) to 3.3 (95% CI 3.1 to 3.4) admissions/1000 term live births; proportion of potentially avoidable hypoglycaemia admissions (enteral feeds and special care only) decreased (from 12.8% (95% CI 12.1% to 13.4%) to 8.9% (95% CI 8.3% to 9.5%)), time to NNU admission and median length of stay were unchanged between epoch 1 and epoch 2. 46.5% (11 825/25 406) of infants admitted primarily for hypoglycaemia had one or more BAPM hypoglycaemia risk factors. Of infants with hypoglycaemia without BAPM risk factors, 44% (5990/13 581) had a birth weight above the 90th centile or between the 2nd centile and the 9.9th centile.

**Conclusions:**

The publication of a national framework for term hypoglycaemia management was associated with a reduction in term NNU admission rates without delays in admission time or increased length of stay. One in two infants admitted for hypoglycaemia had no BAPM risk factors. Future research should explore birth weight between the 2nd centile and the 9.9th centile and above the 90th centile as additional factors that may further enhance hypoglycaemia risk stratification.

**WHAT IS ALREADY KNOWN ON THIS TOPIC**

- Neonatal hypoglycaemia is a leading cause of term infant admission to neonatal units (NNUs).
- The British Association of Perinatal Medicine (BAPM) Framework of Practice ‘Identification and Management of Neonatal Hypoglycaemia’, published in April 2017, identified at-risk infants requiring glucose monitoring and altered the treatment threshold from 2.6 mmol/L to 2.0 mmol/L, accepting lower levels of glucose before clinical intervention and recommended the use of buccal dextrose gel outside of the NNU.

**WHAT THIS STUDY ADDS**

- We observed a reduction in term admissions to the NNU primarily for hypoglycaemia in the 36 months following publication of the BAPM hypoglycaemia framework (April 2017) equivalent to approximately 1300 fewer NNU admissions in England and Wales per year.
- We did not find changes to the interval between birth and admission to NNU or the length of admission for infants admitted for hypoglycaemia in the 36 months before and after publication of the BAPM framework.
- Over half of infants admitted to NNU for hypoglycaemia during 2012–2020 had no BAPM risk factors for hypoglycaemia. Of these infants, 29% were small for gestational age (SGA) with birth weight between the 2nd centile and the 9.9th centile, and 15% were large for gestational age (LGA) with birth weight above the 90th centile.

**HOW THIS STUDY MIGHT AFFECT RESEARCH, PRACTICE OR POLICY**

- Our findings suggest that the changes recommended in the BAPM framework did not increase adverse short-term outcomes.
- Future revisions to the framework should be informed by research that explores whether adding LGA and SGA as risk factors would facilitate earlier intervention and further reduce term NNU admissions for hypoglycaemia.

## Introduction

 Neonatal hypoglycaemia is a leading cause of term admission to neonatal units (NNUs), accounting for approximately 1 in 10 term NNU admissions.^1^ Infants requiring admission to NNU have usually not responded to interventions on the postnatal ward and require assisted enteral feeding with a nasogastric tube or intravenous glucose. Prompt interventions are necessary to avoid hypoglycaemic seizures and brain injury, which can lead to long-term neurodevelopmental impairment.^2^ Early screening, identification and intervention can prevent NNU admissions, separation of mother and baby, and additional workload to neonatal and maternity service, which have cost implications.

The blood glucose definition of hypoglycaemia varies across practice and the literature. Some guidelines define neonatal hypoglycaemia as a blood glucose of <2.6 mmol/L.^3^ Similarly, the risk factors that warrant active monitoring of blood glucose vary across international guidelines.^4^ The British Association of Perinatal Medicine (BAPM) published the Framework of Practice, ‘Identification and Management of Neonatal Hypoglycaemia in the Full Term Infant’, with recommendations on identification and management of term infants at risk of impaired metabolic adaptation and hypoglycaemia in 2017.^5^ This framework aimed to reduce variation in clinical practice and NNU admission rates for hypoglycaemia in term infants (born ≥37 weeks’ gestation). The BAPM framework recommends targeted hypoglycaemia screening for infants with the following risk factors: infants of diabetic mothers, infants of mothers taking beta-blockers and infants with fetal growth restriction (birth weight <2nd centile). The framework altered the treatment threshold for neonatal hypoglycaemia from 2.6 mmol/L to 2.0 mmol/L and introduced 40% buccal dextrose gel as the first-line management of hypoglycaemia outside of NNUs alongside feeding support. Although dextrose gel may be most efficient in mild hypoglycaemia, there is emerging evidence to suggest that it may also reduce NNU admissions.^6–8^ The framework recommends that infants with more than two measurements of blood glucose <2.0 mmol/L with a risk factor for impaired metabolic adaptation should be reviewed by the neonatal team. Any baby with a blood glucose value <1.0mmol/L at any time or clinical signs consistent with hypoglycaemia require urgent review and admission to the neonatal unit for investigation and treatment. The impact of this framework on the rate of admissions to NNU for hypoglycaemia management in the UK is unknown.

We aimed to use real-world data to evaluate the impact of the 2017 BAPM Framework on (1) Admission rates to NNUs primarily for hypoglycaemia and (2) Short-term proxy measures for safety, as well as describe perinatal risk factors for hypoglycaemia and identify potential opportunities to reduce term neonatal hypoglycaemia admissions.

## Methods

This was a retrospective cohort study using prospective, de-identified, routinely recorded neonatal electronic health record data from the National Neonatal Research Database (NNRD, Research Ethics Service approval (10 /H0803/151)). The NNRD holds data on all infants admitted to National Health Service (NHS) NNUs in England and Wales since 2012. Formal comparison with data from a multicentre, randomised controlled trial has confirmed high accuracy (>95%) of NNRD data.^9^ Denominator data were ascertained from population-level Office of National Statistics (ONS) England and Wales term live birth data (infants born ≥37 weeks’ gestation).^10^ The study was prospectively registered (Clinical Trials registration: NCT05015049). We report in line with the Reporting of studies Conducted using Observational Routinely-collected Data guidelines.^11^

### Study population

Infants born between 1 January 2012 and 31 December 2020 at ≥37 weeks’ completed gestation (term infants) admitted to an NHS NNU in England or Wales were eligible for inclusion in this study. In keeping with previous studies, we excluded infants with a congenital abnormality (online supplement 1), those with incongruent survival data (recorded as survived to discharge but with a recorded cause of death), those with implausible birth weight Z-scores (≤4 or ≥4) and those who had not been discharged at the time of data extraction.^12–14^ The number of infants excluded from the NNRD study population was also removed from the population-level ONS denominator for all analyses. Birthweight *Z*-scores were calculated with UK90 reference data using LMS2z function (Super Imposition by Translation and Rotation Growth Curve Analysis (SITAR) package).^15^ We did not include infants presenting to emergency departments or admissions to paediatric services secondary to hypoglycaemia as these admissions are not captured in the NNRD.

### Outcomes

Maternal and neonatal variables were extracted from antenatal, delivery and neonatal NNRD data items. The primary outcome was the admission rate of term infants to NNU primarily for hypoglycaemia per 1000 term live births (ONS denominator data) in England and Wales. Admission primarily for hypoglycaemia was defined as infants with hypoglycaemia recorded as their primary reason for admission on their first NNU admission episode. Secondary outcomes included time from birth to admission, admission blood glucose, length of stay in NNU and resource use, neonatal seizures, severe brain injury and death during NNU stay.^16 17^ Potentially avoidable hypoglycaemia admissions were defined as admitted infants who received enteral feeding in the special care level of care only and no additional interventions in keeping with previous studies that informed the Avoiding Term Admissions Into Neonatal units (ATAIN) programme.^1^ See online supplement 2 for outcome definitions and extraction procedure.

### Risk factors for neonatal hypoglycaemia

Clinical risk factors were defined as per the BAPM Framework: infants of diabetic mothers; infants with fetal growth restriction (birth weight <2nd centile adjusted for gestational age and sex); infants whose mothers have taken beta-blockers (NNRD maternal hypertensive disorder of pregnancy codes and antihypertensive agent exposure at delivery were used as a proxy measure, as reliable maternal medication data are not available in the NNRD).^4 5 18^ Maternal hypertensive disorder of pregnancy codes were *gestational hypertension, pre-eclampsia*, *chronic hypertension* and *haemolysis, elevated liver enzymes and low platelets syndrome* as per previous studies.^12 13^ Additional risk factors include large for gestational age (LGA, birth weight >90th centile), small for gestational age (SGA, birth weight <10th centile) and low temperature (temperature <36.5°C).^19–22^ Outcomes are defined in online supplement 2.

### Statistical methods

Analyses were carried out in R (V.4.4.1).^23^ We compared baseline characteristics descriptively and report the annual admission rate per 1000 term livebirths (ONS denominator). We compared mean annual admission rate primarily for hypoglycaemia in the 36 months before and after the publication of the BAPM Framework. We employed an interrupted time series analysis (ITSA) with a generalised least squares model.^24^ Secondary analysis compared clinical outcomes and potentially avoidable admissions between epochs using logistic regression or Fisher’s exact test, as appropriate. The full statistical plan is presented in online supplement 3.

## Results

We identified 299 961 infants born ≥37 weeks’ gestation admitted to NNUs in England and Wales from 2012 to 2020, which represents 5.3% (95% CI 5.3% to 5.3%) of live births ≥37 weeks recorded by the ONS over this period. After exclusions, 284 646 infants were included in our analysis (online supplement 1).

### Term NNU admissions primarily for hypoglycaemia compared with infants admitted for other reasons

25 406 term infants were admitted to an NNU in England and Wales between 2012 and 2020 primarily for hypoglycaemia, representing 0.4% (95% CI 0.4% to 0.4%) of ONS term live births and 8.5% (95% CI 8.4% to 8.6%) of all term admissions recorded in the NNRD during the study period. Antenatal, delivery and infant characteristics are shown in table 1. Among infants admitted primarily for hypoglycaemia, 63.5% (16 126/25 406) were male and 30.9% (7843/25 406) had a birth weight <2nd centile or >90th centile compared with 58.5% male and 15.0% birth weight <2nd centile or >90th centile in infants admitted for other reasons (table 1). A similar number of mothers were primiparous across both groups (table 1). The proportion of infants exposed to a hypertensive disorder of pregnancy, maternal diabetes or gestational diabetes, induction of labour and emergency caesarean section was higher in the hypoglycaemia admission group compared with infants admitted for other reasons (table 1).

**Table 1:** Baseline characteristics, resource use, interventions required and short-term outcomes for infants admitted to neonatal units for the primary reason of hypoglycaemia compared with those whose primary reason for admission was not hypoglycaemia

	Hypoglycaemia as the primary reason for admission	Other primary reason for admission
*N*	25 406	259 240
*Birth characteristics*		
*Birth year*		
2012–2014, n (%)	9941 (39.1%)	83 486 (32.2%)
2015–2017, n (%)	9675 (38.1%)	91 645 (35.4%)
2018–2020, n (%)	5790 (22.8%)	84 109 (32.4%)
Gestation age at delivery, median weeks (IQR)	38 (37, 39)	39 (38, 40)
Male, n (%)	16 126 (63.5%)	151 737 (58.4%)
Female, n (%)	9261 (36.5%)	107 317 (41.4%)
Missing, n (%)	19 (<1%)	186 (<1%)
Birth weight		
Birthweight centile, median (IQR)	32 (6, 79)	49 (23, 75)
Birthweight centile <10th, n (%)	8204 (32.3%)	31 794 (12.3%)
Birthweight centile <2nd, n (%)*	3292 (13.0%)	9102 (3.5%)
Birthweight centile 2nd to 9.9th, n (%)	4912 (19.3%)	22 692 (8.8%)
Birthweight centile >90th, n (%)	4551 (17.9%)	29 874 (11.5%)
Missing, n (%)	0	10 (<1%)
Singleton, n (%)	24 030 (94.6%)	252 348 (97.3%)
Multifetal, n (%)	1370 (5.4%)	6768 (2.6%)
Missing, n (%)	6 (<1%)	124 (<1%)
*Parity (maternal*)		
Primiparous, n (%)	9520 (37.5%)	91 991 (35.5%)
Missing, n (%)	3868 (15.2%)	40 777 (15.7%)
Maternal age, median years (IQR)	31 (27, 35)	31 (26, 35)
Missing, n (%)	555 (2.2%)	6384 (2.5%)
*Ethnicity (maternal*)		
White, n (%)	14 872 (58.5%)	157 214 (60.7%)
Asian, n (%)	3166 (12.5%)	26 372 (10.2%)
Black, n (%)	1281 (5.0%)	12 278 (4.7%)
Mixed, n (%)	276 (1.1%)	3310 (1.3%)
Other, n (%)	437 (1.7%)	5357 (2.1%)
Missing, n (%)	5374 (21.2%)	54 709 (21.1%)
IMD decile (maternal), median (IQR)	4 (2, 7)	4 (2, 7)
Most deprived deprivation quintile (maternal), n (%)	6649 (26.2%)	68 587 (26.5%)
Hypertensive disorder of pregnancy, n (%)*	3237 (12.7%)	11 626 (4.5%)
Diabetes, n (%)*	2323 (9.1%)	4196 (1.6%)
Gestational diabetes, n (%)*	4787 (18.8%)	13 877 (5.4%)
*Antenatal steroids*		
Complete, n (%)	1637 (6.4%)	10 377 (4.0%)
Incomplete, n (%)	82 (<1%)	696 (<1%)
None given, n (%)	13 697 (53.9%)	118 551 (45.7%)
*Onset of labour*		
Spontaneous, n (%)	9165 (36.1%)	126 475 (48.8%)
Induced, n (%)	8741 (34.4%)	66 629 (25.7%)
Not in labour, n (%)	4641 (18.3%)	35 536 (13.7%)
Unknown, n (%)	2859 (11.3%)	30 600 (11.8%)
*Mode of delivery*		
SVD, n (%)	8059 (31.7%)	95 924 (37.0%)
Instrumental vaginal, n (%)	2952 (11.6%)	39 133 (15.1%)
ELCS, n (%)	293 (1.2%)	2310 (<1%)
EMCS, n (%)	8149 (32.1%)	66 762 (25.8%)
Apgar Score <7 at 5 mins, n (%)	505 (2.0%)	29 366 (11.3%)
Admission time, median hours from birth (IQR)	8.6 (5.1, 14.7)	2.6 (0.6, 15.8)
Admission temperature, median °C (IQR)	36.7 (36.4, 36.9)	36.8 (36.5, 37.1)
Admission temperature 36.5–37.5°C, n (%)	17 485 (68.8%)	176 746 (68.2%)
Admission temperature >37.5°C, n (%)	339 (1.3%)	14 789 (5.7%)
Admission temperature <36.5°C, n (%)	7110 (28.0%)	48 785 (18.8%)
Missing admission temperature, n (%)	472 (1.9%)	18 920 (7.3%)
**Admission glucose**		
Admission glucose ≥2.6 mmol/L, n (%)	5095 (20.1%)	142 872 (55.1%)
Admission glucose ≥2.0 mmol/L and <2.6 mmol/L, n (%)	11 268 (44.4%)	159 210 (61.4%)
Admission glucose 1.0–2.0 mmol/L, n (%)	9540 (37.6%)	10 716 (4.1%)
Admission glucose <1.0 mmol/L, n (%)	1951 (7.7%)	2663 (1.0%)
Missing, n (%)	2647 (10.4%)	86 651 (33.4%)
**Neonatal unit resource use**		
*Highest level of care received*		
Intensive care	2108 (8.3%)	37 760 (14.6%)
High dependency	1943 (7.6%)	68 169 (26.3%)
Special care	21 807 (85.8%)	191 069 (73.7%)
Total length of stay, median days (IQR)	4 (2, 7)	4 (2, 7)
*Interventions in NNU*		
Received mechanical ventilation or NIV	2021 (8.0%)	102 821 (39.7%)
Received intravenous fluids	16 151 (63.6%)	146 857 (56.6%)
Received parenteral nutrition	636 (2.5%)	9388 (3.6%)
Central venous line	1782 (7.0%)	22 971 (8.9%)
Potentially avoidable admissions†	3147 (12.4%)	7863 (3.0%)
**Short-term neonatal outcomes**		
Died	12 (<1%)	1516 (<1%)
Jaundice	3207 (12.6%)	34 233 (13.2%)
Severe brain injury	364 (1.4%)	14 812 (5.7%)
Seizure	242 (1.0%)	7824 (3.0%)
Late-onset sepsis	17 (<1%)	268 (<1%)
Severe NEC	19 (<1%)	68 (<1%)

* British Association of Perinatal Medicine risk factors for hypoglycaemia: infants with fetal growth restriction - birth weight <2nd centile adjusted for gestational age and sex; infants of mothers with diabetes; maternal hypertensive disease used as a proxy measure for exposure to beta-blockers. Risk factors not mutually exclusive.

† Defined as no mechanical ventilation, no non-invasive ventilation, no central venous or arterial line, no peripheral venous line, no percutaneous venous line, no intravenous dextrose given.

ELCS, elective caesarean section; EMCS, emergency caesarean section; IMD, Index of Multiple Deprivation; NEC, necrotising enterocolitis; NIV, non-invasive ventilation; NNU, neonatal unit; SVD, spontaneous vaginal delivery.

Infants admitted for hypoglycaemia mainly received special care (32 147/36 770, 87%), with a smaller proportion receiving high dependency or intensive care level care (table 1). 1782 (7.0%) infants admitted to NNU primarily for hypoglycaemia had a central venous line and 636 (2.5%) received parenteral nutrition (table 1). Overall, the group of infants admitted primarily for hypoglycaemia were less unwell with higher Apgar Scores and required less resuscitation and support while in the NNU compared with the group of infants admitted for other reasons (table 1). Among infants admitted for hypoglycaemia, 364 babies (1.4%) had severe brain injury, 242 (1.0%) had seizures and 12 died (<0.1%) (table 1).

### Changes in term NNU admission rates primarily for hypoglycaemia

The annual number of infants and admission rate of infants admitted to NNU primarily for hypoglycaemia per 1000 term live births between 2012 and 2020 is shown in table 2. In the 36 months prior (April 2014 to March 2017) to the publication of the BAPM Framework (epoch 1), the mean annual admission rate to NNU of term infants primarily for hypoglycaemia was 4.9 (95% CI 4.8 to 5.1) admissions per 1000 term live births. This reduced to a mean of 3.3 (95% CI 3.1 to 3.4) admissions per 1000 term live births during the 36 months following publication of the BAPM Framework (May 2017 to April 2020, epoch 2).

**Table 2:** Number of admissions, admission rate, admission and discharge times, admission blood glucose and resource use for term infants (≥37 weeks) admitted primarily for hypoglycaemia across the study period

Birth year	2012	2013	2014	2015	2016	2017	2018	2019	2020
Number of term admissions primarily for hypoglycaemia (n)	3203	3232	3506	3572	3234	2869	2064	1960	1766
Term (≥37 weeks) live births, ONS (n)	728 092	696 856	693 540	696 117	694 497	677 372	655 416	638 554	612 146
Hypoglycaemia admission rate per 1000 term live births (95% CI)	4.39 (4.23–4.54)	4.63 (4.46–4.78)	5.04 (4.88–5.21)	5.13 (4.95–5.29)	4.66 (4.49–4.81)	4.23 (4.07–4.38)	3.14 (3.01–3.28)	3.06 (2.92–3.20)	2.87 (2.74–3.01)
NNU admission and discharge variables in infants admitted primarily for hypoglycaemia									
Admission time in minutes, median (IQR)	528 (302–946)	510 (312–882)	501 (306–850)	528 (323–885)	520 (323–902)	491 (298–843)	502 (290–847)	519 (294–892)	574 (312–906)
Discharge time (postnatal age in days), median days (IQR)	4 (2–7)	4 (2–7)	4 (2–7)	4 (2–6)	4 (2–6)	4 (2–7)	4 (2–7)	4 (3–7)	4 (3–7)
Admission glucose (mmol/L), median (IQR)	2.0 (1.6–2.5)	2.0 (1.6–2.5)	2.0 (1.6–2.5)	2.0 (1.6–2.5)	2.0 (1.5–2.5)	1.9 (1.4–2.4)	1.8 (1.4–2.3)	1.8 (1.3–2.4)	1.9 (1.4–2.5)
Resource use in NNU									
Received nasogastric feeds and no other major intervention including intravenous dextrose, n (%)	518 (16.2%)	445 (13.8%)	550 (14.3%)	442 (12.4%)	397 (12.3%)	290 (10.1%)	176 (8.5%)	183 (9.3%)	146 (8.3%)
95% CI for proportion of infants that received nasogastric feeds and no intravenous dextrose	14.9%–17.6%	12.5%–15.2%	13.1%–15.7%	11.3%–13.7%	11.2%–13.6%	9.1%–11.3%	7.4%–9.7%	8.2%–10.6%	7.2%–9.5%

NNU, neonatal unit; ONS, Office of National Statistics.

**Figure 1:**
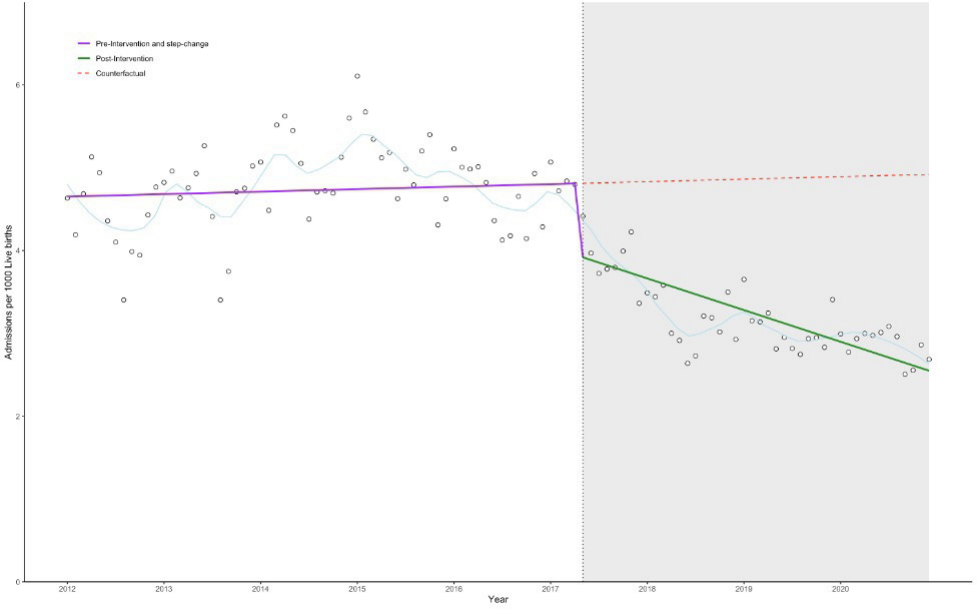
Admission rate to neonatal units for hypoglycaemia in term infants (≥37 weeks) per 1000 term live births in England and Wales, before and after the implementation of the BAPM Framework, with an interrupted time series analysis (ITSA) model. Points = observed admissions. Purple line = modelled rates fitted to the data and immediate step-change. Red dashed line = predicted admissions based on preintervention trends (counterfactual scenario). Blue line = LOESS (locally weighted scatterplot smoothing) line, showing a smoothed trend of the data over time for contextual reference. Grey area = intervention period (c. April 2017). Green line = best fit regression line after the intervention. BAPM, British Association of Perinatal Medicine.

The ITSA model demonstrated that following publication of the BAPM hypoglycaemia framework in April 2017 there was an immediate decrease in admissions primarily for hypoglycaemia of 0.89 per 1000 term live births (figure 1). Admissions for hypoglycaemia continued to reduce by an average of 0.41 admissions per 1000 term live births per year during the 36 months following the publication of the framework (figure 1). The ITSA model showed that the decrease in admissions following the intervention was statistically significant (online supplemental tables 4 and 5).

### Assessing short-term safety using real-world data

Annual median admission blood glucose, time to NNU admission, length of stay and resource use in infants admitted to NNU primarily for hypoglycaemia are shown in table 2. Comparison between epoch 1 and epoch 2 (36 months before and after the publication of the BAPM Framework in April 2017, online supplemental table 6) showed: admission blood glucose was 2.0 (1.5–2.5) mmol/L in epoch 1 and 1.9 (1.4–2.4) mmol/L in epoch 2; median time to admission was 519 min (IQR 317–880; approximately 8.6 hours from birth) in epoch 1 versus 514 min (IQR 292–872) in epoch 2 and median length of stay of 4 days (IQR 2–7 days) remained unchanged between the epochs. There was no increase in the odds of seizures (OR 0.89, 95% CI 0.59 to 1.33), severe brain injury (OR 1.03, 95% CI 0.75 to 1.40) or mortality (OR 0.75, 95% CI 0.15 to 4.83) for infants admitted for hypoglycaemia between epoch 1 and epoch 2 (online supplemental table 6).

### Potentially avoidable term admission primarily for hypoglycaemia

Across the whole study period (2012–2020), 12.1% (3066/25 406) of term NNU hypoglycaemia admissions were deemed potentially avoidable as infants received enteral (nasogastric) feeds only without intravenous dextrose or other major intervention. The annual count of avoidable admissions decreased over the study period, as did the proportion of all hypoglycaemia admissions classified as avoidable, from 12.8% (95% CI 12.1% to 13.4%) in epoch 1 to 8.9% (95% CI 8.3% to 9.5%) in epoch 2.

### Risk factors for hypoglycaemia in term infants according to the BAPM Framework

**Figure 2:**
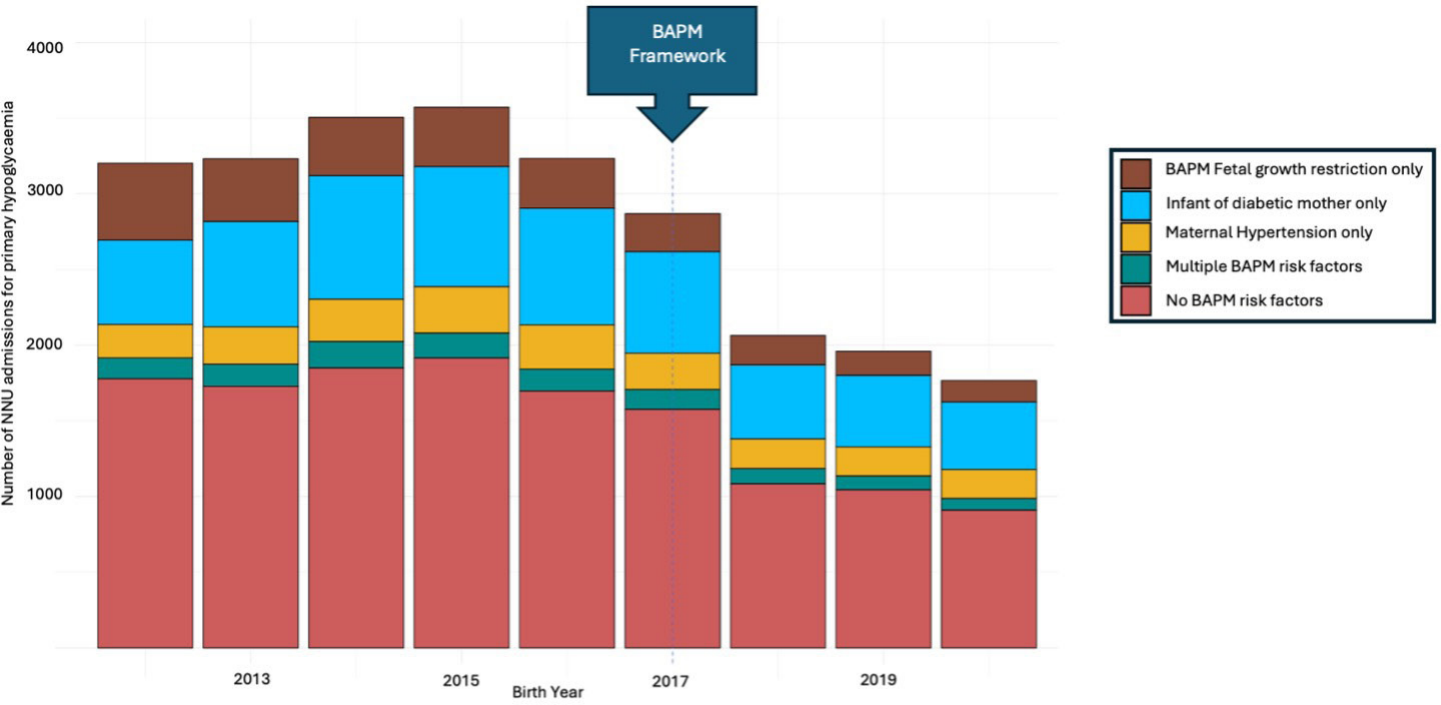
Trends in risk factors for hypoglycaemia in infants admitted primarily for hypoglycaemia born ≥37 weeks’ gestation. BAPM, British Association of Perinatal Medicine; NNU, neonatal unit.

**Figure 3:**
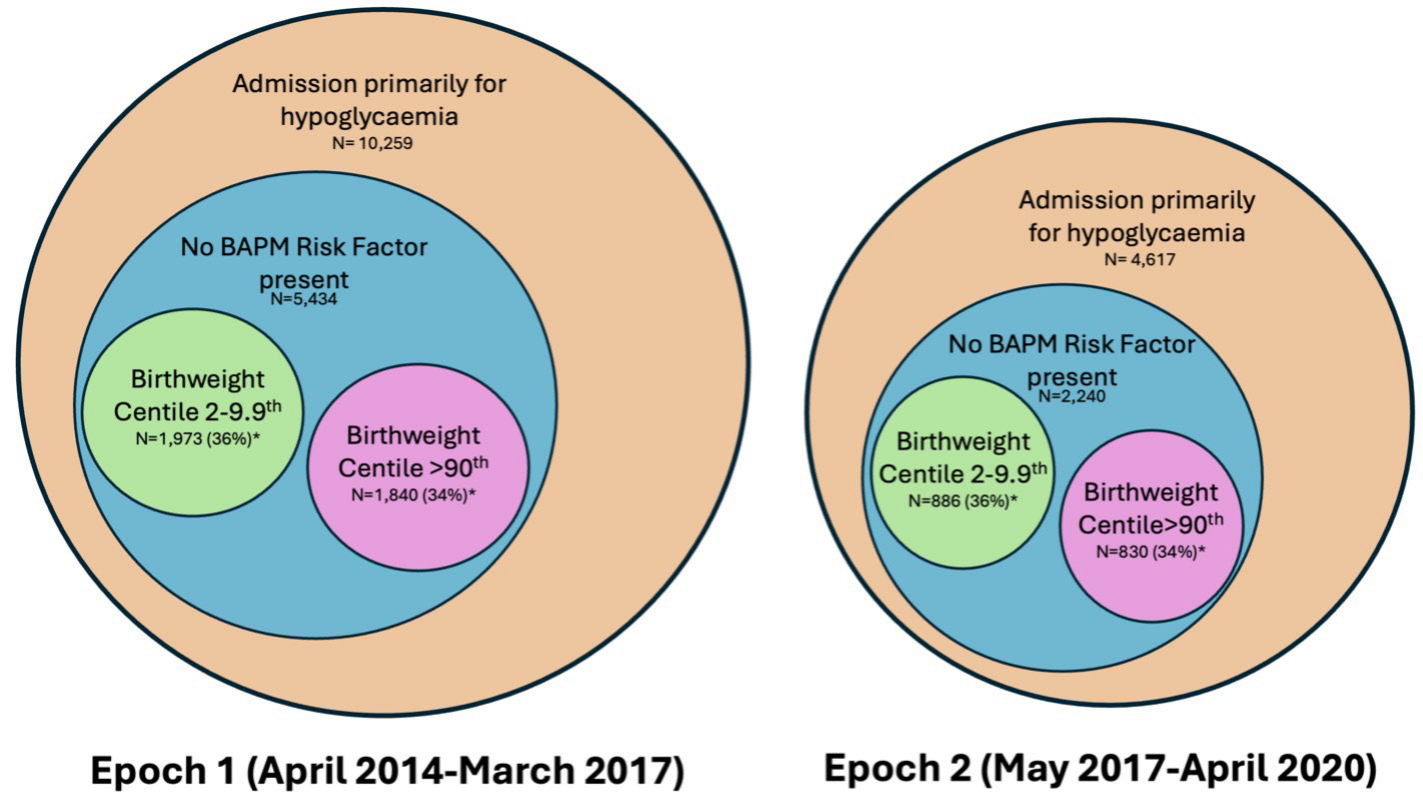
Euler diagrams illustrating the proportion of infants admitted primarily for hypoglycaemia with and without BAPM risk factors recorded during epoch 1 (April 2014 to March 2017) and epoch 2 (May 2017 to April 2020). For those with no BAPM risk factor, we present the proportion of babies with birth weight 2nd centile to 9.9th centile and >90th centile (using infants with no BAPM risk factors as denominator). Orange = BAPM risk factor present, blue = no BAPM risk factor, pink = birth weight >90th centile, green = birth weight 2nd to 9.9th centile, *denominator for proportion is number of infants with no BAPM risk factors. BAPM, British Association of Perinatal Medicine.

Of the 25 406 term infants admitted to NNU primarily for hypoglycaemia, 5713 (22.5%) were exposed to maternal diabetes only, 2780 (10.9%) were growth restricted only (birth weight <2nd centile), 2163 (8.5%) were exposed to maternal hypertension only (proxy for beta-blocker exposure) and 1169 (4.5%) had multiple (two or more) risk factors for hypoglycaemia (figure 2). More than half of the admitted infants (13 581; 53.5%) had no BAPM risk factors for hypoglycaemia; comparing epoch 1 to epoch 2, the proportion of babies with no BAPM risk factors for hypoglycaemia remained unchanged at just around half of all admissions (figure 3, online supplemental table 6).

### Other risk factors for hypoglycaemia in term infants

Of the 25 406 term infants admitted to NNU primarily for hypoglycaemia, 4912 (19.3%) had a birth weight between the 2nd and the 9.9th centile and 4551 (17.9%) had a birth weight above the 90th centile. Exploring the subgroup of infants admitted primarily for hypoglycaemia with no BAPM risk factors (n=13 581), 3908 (28.8%) infants had a birth weight between the 2nd and the 9.9th centile and 2082 (15.3%) had a birth weight above the 90th centile (figure 3, online supplemental table 7). Admission temperatures were similar across both groups (online supplemental table 7). In epoch 1, 5434 (53%) of the infants had BAPM risk factors versus 2440 (53%) in epoch 2 (figure 3, online supplemental table 5). Across both epochs, babies with birth weight between the 2nd and the 9.9th centile or above the 90th centile comprised 70% (3813/5434—epoch 1; 1716/2440—epoch 2) of the babies with no BAPM risk factors for hypoglycaemia (figure 3, online supplemental table 6).

## Discussion

### Main findings

Between 2012 and 2020 over 1 in 20 term liveborn infants were admitted to NNU annually, with over 1 in 12 NNU admissions being primarily for hypoglycaemia.Time-series analysis demonstrated an association between a reduction in NNU term admissions for hypoglycaemia and the publication of the BAPM hypoglycaemia framework (April 2017).

The BAPM framework identified at-risk infants requiring glucose monitoring and altered the treatment threshold for neonatal hypoglycaemia from 2.6 mmol/L to 2.0 mmol/L, accepting lower levels of glucose before clinical intervention and recommended the use of buccal dextrose gel outside of the NNU. Therefore, our finding of a lower rate of NNU hypoglycaemia admissions may be a result of earlier identification, intervention and avoidance of deterioration and admission. Reassuringly, we did not observe an increase in time to admission, neonatal length of stay, neonatal seizures or severe brain injury, which could have occurred if reducing the acceptable blood glucose level led to delays in admission and treatment or increase in harm (eg, clinical compromise). Furthermore, the finding that avoidable admissions (requiring only enteral feeds) reduced following BAPM Framework publication suggests effective infant hypoglycaemia risk stratification by admitting infants in genuine need of medical intervention. Over half of all term NNU hypoglycaemia admissions had no BAPM risk factors. Importantly, 29% of infants with no BAPM risk factors had a birth weight between the 2nd and 10th centile and 15% had a birth weight above the 90th centile.

### Strengths and limitations

This study has several strengths, including the availability of data from a large national population cohort across a 9-year period. Detailed NNRD data enabled identification of NNU hypoglycaemia admissions using the variable ‘primary reason for neonatal admission’. Furthermore, the extensive data set including daily data enabled detailed characterisation of the study population.

However, we acknowledge the limitations of this study. First, as these are routinely recorded clinical data, not collected specifically for research, it is possible that the primary admission reason may be erroneous. However, the finding that infants admitted primarily for hypoglycaemia are smaller, induced, born in better condition and need less interventions compared with those admitted for other reasons, provides confidence in the plausibility and accuracy of the data. Second, in the absence of data on maternal medications and beta-blocker use, we used maternal pregnancy or chronic hypertension as a surrogate risk factor for beta-blocker exposure. We acknowledge this may over-report the proportion of infants with BAPM risk factors given that other non-beta-blocker medications such as calcium channel antagonists are also used for the management of hypertension in pregnancy.

A third limitation is the absence of denominator data on ‘at risk’ infants born given that the NNRD only includes NNU admissions. National data for mothers and infants with risk factors (eg, birthweight centile, maternal hypertensive medication or diabetes) cared for outside of the NNU are not available. As such, we were unable to determine what proportion of LGA (birth weight >90th centile) and SGA (birth weight <10th centile) infants develop hypoglycaemia. This information is needed to evaluate the potential implications of changes to the BAPM risk factors on the workload of maternity staff. Furthermore, infants admitted for reasons other than primary hypoglycaemia are sick, term infants requiring care rather than a valid control group for comparison. Regarding the assessment of short-term safety of the changes recommended in the BAPM framework, adverse events such as severe brain injury secondary to hypoglycaemia are relatively rare events. Therefore, while we did not observe any safety signals in these data, we have limited power to detect changes and recommend that ongoing surveillance of potential adverse effects of raising the hypoglycaemia treatment threshold is warranted. In addition, we were unable to report long-term neurodevelopmental outcomes or data on re-admissions following discharge, which are also important safety measures.

### Clinical relevance and future research

There is wide variation in screening criteria and management for neonatal hypoglycaemia internationally.^4^ Current American and Canadian infant hypoglycaemia guidelines recognise LGA (birth weight >90th centile) and SGA (birth weight <10th centile) as hypoglycaemia risk factors warranting screening. Our finding that 29% of all infants admitted for primary hypoglycaemia without BAPM risk factors are born with a birth weight on the 2nd centile to the 9.9th centile and 15% are born LGA (birth weight >90th centile) supports the need for future research to evaluate the potential impact of these additional risk factors on term NNU hypoglycaemia admissions and maternity workload. Targeted screening of these groups may facilitate earlier intervention and contribute to a reduction in neonatal admissions. Of note, inclusion of LGA in BAPM guidelines has been discussed in a recent consultation on the guidelines, but authors concluded there is insufficient evidence to recommend inclusion at present, acknowledging undiagnosed maternal diabetes is outside of the scope of the guideline.^25^

Furthermore, our finding that a higher proportion of NNU hypoglycaemia admissions (vs admitted for other reasons) are born to mothers with pregnancy hypertension raises the question of whether hypertensive disorders themselves or specific drug exposure drive neonatal hypoglycaemia, an area of ongoing uncertainty.^26–28^ Further research is needed to determine optimal maternal antihypertensives for both mother and baby.^28^ Similarly, exposure to maternal diabetes was much more common in NNU hypoglycaemia admissions and supports the need for continued effort in prevention and optimal management of maternal diabetes.

## Conclusions

In this retrospective cohort study, we found a reduction in infant NNU term hypoglycaemia admissions, associated with publication of the BAPM Framework on Identification and Management of Hypoglycaemia in term infants.^5^ This reduction was not accompanied by delayed or longer admission to NNU or increases in short-term adverse outcomes such as seizures or severe brain injury. Further investigation of the impact of BAPM guidelines on re-admissions following discharge and long-term developmental outcomes is required to definitively assess safety of the recommendations. Our finding that over half of term infants admitted to NNU for hypoglycaemia did not have a BAPM-recognised risk factor for hypoglycaemia emphasises the need to explore additional screening criteria. Future studies should explore where addition of LGA and SGA (2nd centile to 9.9th centile) infants to the BAPM Framework may further reduce term admissions to NNU for hypoglycaemia management.

## Supplementary material

10.1136/archdischild-2025-328872 online supplemental file 1

## Data Availability

Data may be obtained from a third party and are not publicly available.
