# Supplementary material for: Term neonatal admissions for hypoglycaemia in England and Wales, 2012–2020: a population-based study using the National Neonatal Research Database
Source: Arch Dis Child Fetal Neonatal Ed. 2025 Dec 3;111(4):e328872. doi: 10.1136/archdischild-2025-328872 (PMC13479278; doi:10.1136/archdischild-2025-328872)
Supplement: Supplementary file 1 [file fetalneonatal-111-4-s001.pdf]

Supplementary Appendix

Contents

Supplement 1: Study flow chart	Page 2
Supplement 2: Outcomes, risk factors and covariates: definitions and NNRD extraction procedures	Page 3
Supplement 3: Statistical Plan	Page 13
Supplement Table 4: Interrupted Time Series Model – Term infants	Page 15
Supplement Table 5: Interrupted Time Series Model – Term infants model Diagnostics	Page 15
Supplement Table 6: Risk factors for hypoglycaemia and outcomes for infants admitted to neonatal units for hypoglycaemia during epoch one (April 2014-March 2017) and epoch two (May 2017-April 2020).	Page 16
Supplement Table 7: Baseline characteristics for infants admitted to neonatal units for hypoglycaemia with BAPM Risk Factors compared to those without BAPM Risk Factors	Page 17
Supplement Table 8: UK Neonatal Collaborative units in England and Wales	Page 19

Supplement 1: Study flow chart

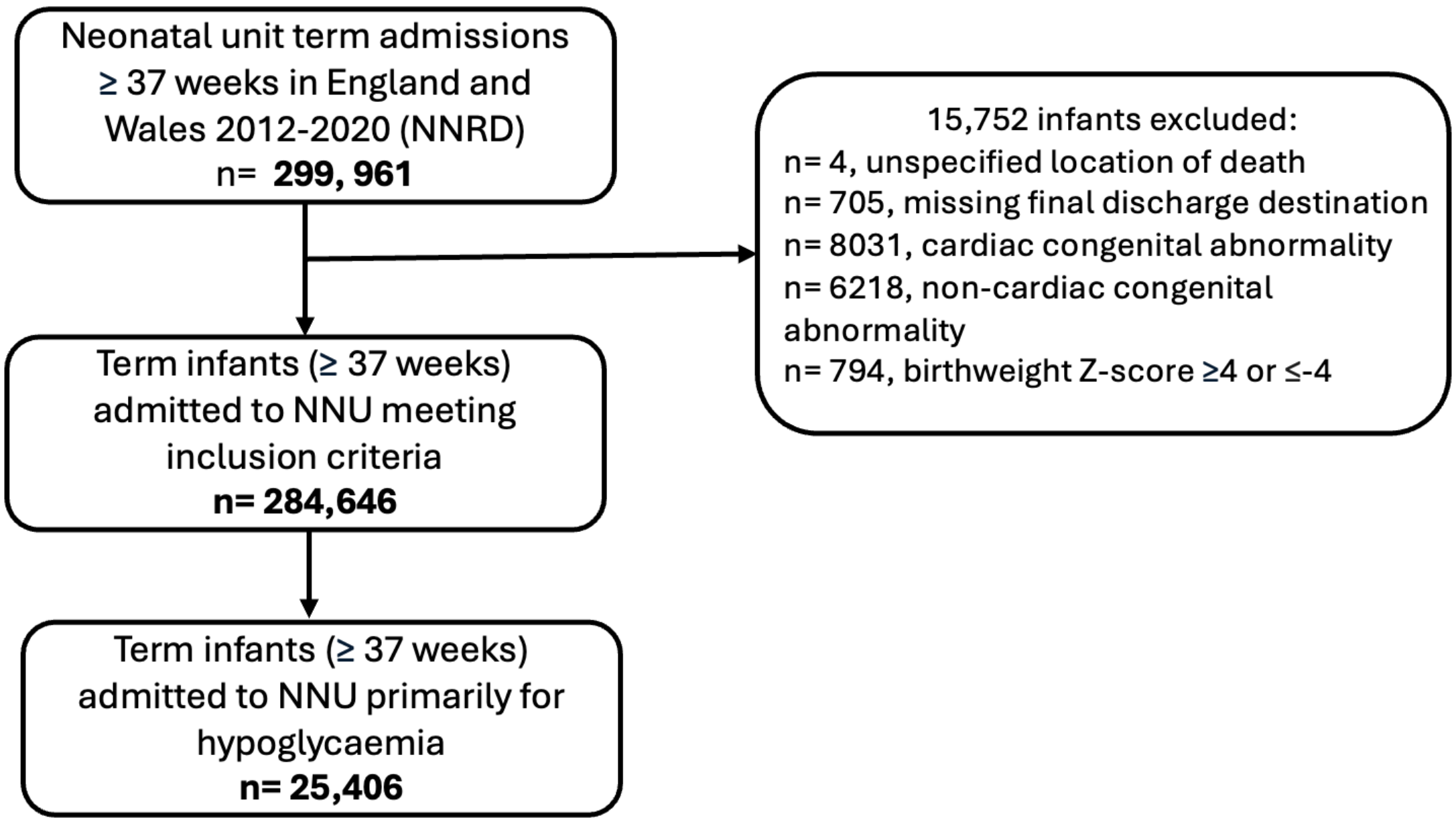

Supplement 2: Outcomes, risk factors and covariates: definitions and NNRD extraction procedures

Maternal and neonatal variables were extracted from antenatal, delivery and neonatal NNRD data items.

Secondary outcomes included time from birth to admission, admission blood glucose measurement, length of stay in the neonatal unit and resource use (intravenous fluids, central line, NG feeds) for infants admitted to NNU primarily for hypoglycaemia, neonatal seizures, severe brain injury (any of hypoxic ischaemic encephalopathy, intracranial haemorrhage, central nervous system infection, kernicterus or seizures as previously defined in NNRD studies, and death during neonatal unit stay.

Potentially avoidable hypoglycaemia admissions were defined as admitted infants who received enteral feeding in special care (SC) level of care only and no additional interventions (no respiratory support, no central venous or arterial line, no peripheral venous line, no percutaneous venous line, no intravenous dextrose). Daily level of care was determined according to Health Resource Group (HRG) definitions based on category of care received.

Established clinical risk factors for hypoglycaemia were defined in the NNRD as per the BAPM Framework: infants of diabetic mothers; infants with fetal growth restriction; infants whose mothers have taken beta blockers (NNRD maternal hypertensive disorder of pregnancy codes and antihypertensive agent exposure at delivery were used as a proxy measure, as reliable maternal medication data is not available in the NNRD and the beta-blocker labetalol is the most common treatment for hypertension in pregnancy). Procedures for extraction of risk factors for neonatal hypoglycaemia are detailed below:

**Variable**	**NNRD extraction procedure**
Chronic hypertension (CHT)	*Antenatal variables*<br>Variable: ProblemsMedicalMother<br>Coding: Dichotomous, code 12 (chronic hypertension) = TRUE, other codes = FALSE.
Gestational hypertension (GH)	*Antenatal variables*<br>Variable: *ProblemsDuringPregnancy*<br>Coding: Dichotomous, code 30 (Pregnancy induced hypertension) = TRUE, other codes = FALSE.

Pre-eclampsia (PET)	*Antenatal variables*<br>Variable: *ProblemsDuringPregnancy*<br>Coding: Code 31 (Pre-eclampsia) = TRUE<br><br>AND/OR<br><br>*Discharge diagnosis table*<br>Coding: TRUE if any of the following codes:<br>15389 (mild preeclampsia)<br>15390 (moderate preeclampsia)<br>15391 (severe preeclampsia)
Haemolysis, elevated liver-enzymes and low platelets syndrome (HELLP)	*Antenatal variables*<br>Variable: *ProblemsDuringPregnancy*<br>Coding: Dichotomous, code 32 (maternal HELLP) = TRUE, other codes = FALSE.
Hypertensive disorder not otherwise specified (NOS)	*Labour and delivery*<br>Variable: Drugs in Labour<br>Coding: Dichotomous, code 18 (antihypertensive) = 1, other codes = 0.
Survival to discharge from neonatal unit	WHERE<br>Discharge destination at final admission = 'Died'<br>THEN (0)<br><br>0 = Died<br>1 = Survived to discharge<br>9 = Unknown
Survival to discharge from neonatal care without comorbidity	WHERE<br><br>Survival == 1 AND<br><br>IF Treated_ROP = 0 AND BPD = 0 AND<br>SevereNEC = 0 AND Brain injury = 0<br>THEN<br><br>0 = Died<br>1 = Survival with comorbidities<br>2 = Survival no comorbidities
Fetal growth restriction *(Risk factor for indicated preterm birth)*	*Antenatal variables*<br>Variable: *ProblemsDuringPregnancy*<br>Coding: TRUE if any of the following codes:<br>"Intrauterine growth restriction" (10)<br>"Poor biophysical profile" (11)<br><br>AND/OR

	Discharge diagnosis table<br>Variables: *PrincipalDiagnosisAtDischarge* and text in diagnosis table have any of the following terms (== TRUE):<br><br>‘15510’: ‘INTRAUTERINE GROWTH RESTRICTION (IUGR)’<br><br>‘1010152’: ‘REVERSED DIASTOLIC FLOW (MATERNAL DOPPLERS)’<br><br>‘15505’: ‘SMALL FOR DATES – NO MALNUTRITION (SGA/IUGR)’<br><br>‘1010151’: ‘ABSENT DIASTOLIC FLOW (MATERNAL DOPPLERS)’<br><br>‘15509’: ‘FETAL GROWTH RETARDATION’<br><br>‘1010295’: ‘SMALL FOR GESTATIONAL AGE’<br><br>AND/OR<br><br>Birthweight centile (UK90) < 3rd centile
Antepartum haemorrhage *(Risk factor for indicated preterm birth)*	*Antenatal variables*<br>Variable: *ProblemsDuringPregnancy*<br>Coding: TRUE if any of the following codes:<br>27 – Placental abruption<br>28 – Placenta praevia<br>29 – Other antepartum haemorrhage
Rupture of membranes and chorioamnionitis *(Risk factor for indicated preterm birth)*	*Antenatal variables*<br>Variable: *ProblemsDuringPregnancy*<br>Coding: TRUE if any of the following codes:<br>18 – Chorioamnionitis<br>19 – Preterm rupture of membranes<br>20 – Prolonged rupture membranes<br>21 – Cervical suture<br>22 – Maternal Gp B Strep
Multi-fetal pregnancy *(Risk factor for indicated preterm birth)*	*Antenatal variables*<br>Fetus number > 1<br><br>OR<br><br>If fetus number missing, use:<br><br>*Antenatal variables*<br>Variable: *ProblemsDuringPregnancy*<br>Coding: TRUE if any of the following codes:<br>17 – Twin to twin transfusion

Other risk factor for indicated preterm birth	*Antenatal variables*<br>Variable: *ProblemsDuringPregnancy*<br>Coding: TRUE if any of the following codes:<br>12 – Reduced fetal movements<br>13 – Oligohydramnios<br>14 – Polyhydramnios<br>16 – Cord problems<br>24 – Other infection<br>25 – Rhesus<br>26 – Other haemolytic disease<br>34 – Cholestasis of pregnancy
Hypoglycaemia diagnosis	The following terms or codes in discharge diagnoses or the diagnosis table:<br><br>'15773': 'HYPERINSULINAEMIC HYPOGLYCAEMIA'<br><br>'15771': 'IATROGENIC NEONATAL HYPOGLYCAEMIA'/'HYPOGLYCAEMIA'/'NEONATAL HYPOGLYCAEMIA'/'OTHER NEONATAL HYPOGLYCAEMIA'<br><br>'500530': 'DRUG INDUCED HYPOGLYCAEMIA'
Treated retinopathy of prematurity	WHERE<br>Surgery for ROP listed OR<br>Cryotherapy, laser therapy, anti-VEGF therapy, other treatment listed in right or left eye<br>OR<br>Avastin given<br>AND<br>GestationWeeks at birth < 32<br>AND<br>FirstBirthWeight < 1500g<br><br>THEN == 1, ELSE == 0
Bronchopulmonary dysplasia	WHERE<br>GestationWeeks at birth < 32 weeks<br>AND<br>Any respiratory support given at WEEK 36 corrected gestational age<br>THEN<br><br>0 = Died before week 36<br>1 = No respiratory support given at 36 weeks<br>2 = Respiratory support at 36 weeks<br>9 = Unknown<br><br>* Where no data for week 36 available due to early discharge, data is taken from the final or penultimate day on the unit
Total length of stay in neonatal unit	SUM (Discharge time – Admission time) for each episode of care<br>In days
Number of days of intensive/high dependency/special care	SUM DAILY RECORDS WHERE<br>'HRG2016' = intensive care / high dependency / special care as appropriate

Congenital abnormality – non cardiac	<u>Coding:</u> TRUE if any of the following codes in Admission or Discharge Diagnosis:<br>15890 Anencephaly<br>15891 Craniorachischisis<br>15892 Iniencephaly<br>15893 Anencephaly and similar malformations<br>15894 Frontal encephalocele<br>15895 Nasofrontal encephalocele<br>15896 Occipital encephalocele<br>15897 Encephalocele of other sites<br>15898 Encephalocele (unknown or unspecified cause)<br>15899 Encephalocele<br>15926 Cervical spina bifida with hydrocephalus<br>15927 Thoracic spina bifida with hydrocephalus<br>15928 Lumbar spina bifida with hydrocephalus<br>15929 Sacral spina bifida with hydrocephalus<br>15930 (unknown or unspecified cause) spina bifida with hydrocephalus<br>15931 Cervical spina bifida without hydrocephalus<br>15932 Thoracic spina bifida without hydrocephalus<br>15933 Lumbar spina bifida without hydrocephalus<br>15934 Sacral spina bifida without hydrocephalus<br>15935 Spina bifida (unknown or unspecified cause)<br>10986 Spina bifida<br>10704 Myelomeningocele (specify site)<br>15936 Spina bifida<br>10741 Oesophageal atresia without distal fistula<br>16195 Atresia of oesophagus without fistula<br>10740 Oesophageal atresia with distal 7rachea-oesophageal fistula<br>16196 Atresia of oesophagus with 7rachea-oesophageal fistula (TOF)<br>16197 Congenital 7rachea-oesophageal fistula without atresia (TOF)<br>10273 Congenital stenosis of the oesophagus<br>16198 Congenital stenosis and stricture of oesophagus<br>16199 Oesophageal web<br>10358 Duodenal atresia / stenosis / web (specify)<br>16212 Congenital absence, atresia and stenosis of duodenum<br>16213 Duodenal atresia / stenosis<br>10605 Jejunal atresia / stenosis (specify)<br>16214 Jejunal atresia / stenosis<br>10541 Ileal atresia / stenosis (specify)<br>16215 Congenital absence, atresia and stenosis of ileum<br>16216 Ileal atresia / stenosis<br>16217 Congenital absence, atresia and stenosis of small intestine<br>16218 Cong absence atresia / stenosis of rectum with fistula<br>10496 High anorectal anomaly with rectourethral fistula<br>10497 High anorectal anomaly with rectovesical fistula<br>10498 High anorectal anomaly with rectovulval fistula<br>10495 High anorectal anomaly with rectocutaneous fistula<br>10494 High anorectal anomaly with rectocloacal fistula<br>10493 High anorectal anomaly with fistula (specify)<br>10499 High anorectal anomaly without fistula

	16219 Cong absence atresia / stenosis rectum without fistula<br>16220 Cong absence atresia / stenosis anus with fistula<br>10636 Low anorectal anomaly with anocutaneous fistula<br>10637 Low anorectal anomaly with anovestibular fistula<br>10638 Low anorectal anomaly with fistula (other specify)<br>10639 Low anorectal anomaly without fistula<br>16221 Cong absence atresia / stenosis anus without fistula<br>10240 Congenital anal stenosis<br>16222 Congen absence atresia / stenosis parts of large intestine<br>16223 Cong abscence atresia / stenosis of large intestine part unspec<br>16224 Congenital absence, atresia and stenosis of large intestine<br>10123 Bilateral renal agenesis<br>16318 Renal agenesis, bilateral<br>16324 Potter's syndrome<br>16327 Polycystic kidney, infantile type<br>10100 Autosomal recessive polycystic kidney – infantile<br>10099 Autosomal dominant polycystic kidney in childhood<br>16328 Polycystic kidney, adult type<br>10066 Adult polycystic kidney disease type I<br>10367 Ectopia vesicae<br>16356 Exstrophy of urinary bladder<br>10854 Posterior urethral valves (PUV)<br>16357 Congenital posterior urethral valves (PUV)<br>16360 Congenital absence of bladder and urethra<br>10008 Absence of bladder<br>10236 Congenital absence of urethra<br>16475 Thanatophoric short stature<br>10246 Congenital diaphragmatic hernia<br>10490 Hernia into the cord<br>16495 Congenital diaphragmic hernia<br>16496 Aplasia of diaphragmic hernia<br>16497 Eventration of diaphragmic hernia<br>16498 Exomphalos Malrotation<br>10395 Exomphalos<br>16499 Gastroschisis<br>16589 Trisomy 21, meiotic nondisjunction<br>16590 Trisomy 21, mosaicism (mitotic nondisjunction)<br>16591 Trisomy 21, translocation<br>16592 Down's syndrome (unknown or unspecified cause)<br>16593 Down's syndrome<br>16594 Trisomy 18, meiotic nondisjunction<br>16595 Trisomy 18, mosaicism (mitotic nondisjunction)<br>16596 Trisomy 18, translocation<br>16597 Edwards' syndrome (unknown or unspecified cause)<br>16598 Trisomy 13, meiotic nondisjunction<br>16599 Trisomy 13, mosaicism (mitotic nondisjunction)<br>16600 Trisomy 13, translocation<br>16601 Patau's syndrome (unknown or unspecified cause)<br>16602 Edwards' syndrome and Patau's syndrome
Congenital abnormality – cardiac	<u>Coding:</u> TRUE if any of the following codes in Admission or Discharge Diagnosis:

	16024 Common arterial trunk (Truncus malformation)<br>10356 Double outlet right ventricle (DORV)<br>16025 Double outlet right ventricle (DORV)<br>16026 Double outlet left ventricle (DOLV)<br>11070 Transposition of the great vessels (TGA)<br>16027 Transposition great arteries (TGA)<br>16028 Double inlet ventricle (DILV)<br>16029 Discordant atrioventricular connection<br>16030 Isomerism of atrial appendages<br>16031 Other cong malforms of cardiac chambers and connections<br>16032 Cong malforms of cardiac chambers and connections unspec<br>16033 Congenital malformations of cardiac chambers and connections<br>16035 Atrium single<br>16036 Ventricle single<br>10097 Atrio-ventricular septal defect (AVSD)<br>16039 Atrioventricular septal defect (AVSD)<br>11043 Tetralogy of Fallot<br>16040 Tetralogy of Fallot<br>16045 Pulmonary valve atresia<br>16046 Congenital pulmonary valve stenosis<br>16047 Congenital pulmonary valve insufficiency<br>16048 Other congenital malformations of pulmonary valve<br>16049 Congenital tricuspid atresia / stenosis<br>16050 Ebstein's anomaly<br>16051 Hypoplastic right heart syndrome<br>16052 Other congenital malformations of tricuspid valve<br>16053 Congenital malformation of tricuspid valve (unknown or unspecified cause)<br>16054 Congenital malformations of pulmonary and tricuspid valves<br>16055 Congenital stenosis of aortic valve (AS)<br>16056 Congenital insufficiency of aortic valve<br>16057 Congenital mitral stenosis (MS)<br>16058 Mitral atresia<br>16059 Hypoplastic left heart syndrome (HLH)<br>16060 Other congenital malformations of aortic and mitral valves<br>16061 Congenital malformation of aortic and mitral valves unspec<br>16062 Congenital malformations of aortic and mitral valves<br>16079 Coarctation of aorta<br>10227 Coarctation of the aorta<br>16080 Hypoplasia of aortic arch<br>16081 Stenosis of aorta (AS)<br>16082 Malformation of aorta<br>16083 Atresia of pulmonary artery<br>16084 Stenosis of pulmonary artery (PS)<br>16086 Other congenital malformations of great arteries<br>16087 Transposition of the great vessels (TGA)<br>11057 Total anomylous pulmonary venous drainage (TAPVD)<br>16092 Total anomalous pulmonary venous connection (TAPVD)

Severe Brain Injury	Severe Brain Injury includes the following diagnosis:<br>**<u>HIE:</u>**<br>Defined as diagnoses of 'Grade 3' or 'Grade 2' HIE from all diagnosis fields in the NNRD<br>OR<br>Therapeutic Hypothermia for 2 or more consecutive days<br>**<u>ICH:</u>**<br>Defined as diagnosis of 'Grade '3' or 'Grade 4' IVH from Cranial scans table in the NNRD<br>OR<br>Any of the following diagnoses in any of the diagnosis fields in the NNRD:<br>• SUBDURAL HAEMORRHAGE (DUE TO BIRTH INJURY)<br>• CEREBRAL HAEMORRHAGE DUE TO BIRTH INJURY<br>• TRAUMATIC INTRAVENTRICULAR HAEMORRHAGE<br>• SUBARACHNOID HAEMORRHAGE<br>• SUBARACHNOID HAEMORRHAGE DUE TO BIRTH INJURY<br>• TENTORIAL TEAR DUE TO BIRTH INJURY<br>• INTRACRANIAL LACERATION AND HAEMORRHAGE DUE TO BIRTH INJURY<br>• INTRACRANIAL HAEMORRHAGE (UNKNOWN OR UNSPECIFIED CAUSE)<br>• INTRACEREBRAL HAEMORRHAGE (TERM INFANT)<br>• INTRAVENTRICULAR HAEMORRHAGE (PERINATAL)<br>• POST-HAEMORRHAGIC HYDROCEPHALUS<br><br>OR<br>Any of the following procedures in any of the procedures fields in the NNRD:<br>• VENTRICULOPERITONEAL OR OTHER VENTRICULAR SHUNT<br>• EXTERNAL VENTRICULAR DRAIN<br>• VENTRICULAR DRAIN WITH RESERVOIR<br>• INSERTION OF VENTRICULAR PERITONEAL SHUNT<br>• INSERTION OF RICKHAM RESERVOIR<br>• INSERTION OF VENTRICULO-ATRIAL CSF SHUNT<br>• INSERTION OF VENTRICULO-PERITONEAL CSF SHUNT<br>• CREATION OF VENTRICULOPERITONEAL SHUNT<br><br>**<u>CNS infection:</u>**<br>Defined as any of the following diagnoses in any diagnosis field within the NNRD:<br>• ENCEPHALITIS<br>• CANDIDA ENCEPHALITIS

	• BACTERIAL MENINGITIS<br>• VIRAL MENINGITIS<br>• MENINGITIS - BACTERIAL (SPECIFIC ORGANISM)<br>Specific options found in NNRD:<br>o MENINGITIS – STREPTOCOCCAL<br>o MENINGITIS – CANDIDA<br>• MENINGITIS - BACTERIAL (UNKNOWN OR UNSPECIFIED CAUSE)<br>• CONGENITAL HERPES<br><br>OR<br>Any entry of any growth from a CSF culture<br>**<u>Cystic PVL (preterm white matter injury):</u>**<br>Defined as being born at less than 37 weeks gestational age AND having any of the following diagnoses in any of the diagnosis field in the NNRD:<br>• CYSTIC PERIVENTRICULAR LEUKOMALACIA<br>OR<br><br>Being born < 37 weeks gestational age and having a diagnosis of PVL from Cranial scans table in the NNRD<br><br>**<u>Kernicterus:</u>**<br><br>Defined as any of the following diagnoses in any of the diagnosis fields within the NNRD:<br>• KERNICTERUS<br>• BILIRUBIN ENCEPHALOPATHY<br><br>**<u>Perinatal Stroke:</u>**<br>Defined as any of the following diagnoses in any of the diagnosis fields within the NNRD:<br>• NEONATAL STROKE<br>• INFARCTION: MIDDLE CEREBRAL ARTERY<br>• CEREBROVASCULAR ACCIDENT<br>• CEREBRAL VENOUS THROMBOSIS<br>• NEONATAL CEREBRAL ISCHAEMIA<br><br>**<u>Seizures:</u>**<br>Defined as any entry of ‘Convulsions’ in the Daily summary table in the NNRD<br><br>Term babies would be considered as having a brain injury where any of the following is detected:<br>• HIE<br>• Intracranial haemorrhage<br>• Perinatal stroke<br>• CNS infection<br>• Kernicterus<br>• Seizures<br><br>Preterm babies would be considered as having a brain injury where any of the following is detected:<br>• Seizures<br>• HIE<br>• Intracranial haemorrhage<br>• Preterm white matter injury

	• Perinatal stroke<br>• CNS infection<br>• Kernicterus

Supplement 3: Statistical Plan

All analyses were carried in R (version 4.4.1).(25) Baseline neonatal and maternal characteristics are reported using descriptive statistics, with counts and proportions and means for categorical and continuous variables respectively, comparing term infants admitted to NNU primarily for hypoglycaemia to term infants admitted for other reasons.

To investigate changes in term admission rates primarily for hypoglycaemia and the impact of publication of the BAPM framework (published April 2017), the annual admission rate per 1000 term livebirths (ONS denominator) is reported. We compared mean annual admission rate primarily for hypoglycaemia in the 36 months prior to publication of the BAPM framework (April 2014-March 2017) to the 36 months post publication of the BAPM framework (May 2017-April 2020).

We further employed an Interrupted Time Series Analysis (ITSA) to evaluate the effect of the BAPM framework on the rate of term admissions to NNU primarily for hypoglycaemia per 1000 term live births. For the ITSA model we calculated the monthly admission rate primarily for hypoglycaemia. The gls() function from the nlme package was fitted to create a generalised least squares model with autocorrelation structure to account for autocorrelation between successive months. This was visualised using ggplot2.

To assess the impact of the BAPM framework on short-term proxy measures for safety, we report the annual:

I. admission blood glucose;
II. time to NNU admission;
III. length of stay;
IV. resource use;
V. rates of death, seizures and severe brain injury during neonatal stay

in infants admitted to NNU primarily for hypoglycaemia between 2012 and 2020 and compared figures from the 36 months prior (epoch1) and 36 post publication (epoch2) of the BAPM framework as described above.

We evaluated changes in rates of seizures, severe brain injury and death by calculating odds ratio with 95% confidence intervals between epoch 1 and epoch 2.

For rare outcomes (<5 events per epoch) we used Fisher's exact test and for more common outcomes we used logistic regression. We further investigated the impact of the BAPM framework on potentially

avoidable term admissions for hypoglycaemia, reporting the annual count and proportion of term NNU hypoglycaemia admissions that were potentially avoidable (defined in 'Outcomes' above), and comparing the 36 months prior and post publication of the BAPM framework.

We present counts and proportions of the BAPM risk factors present in term infants admitted primarily for hypoglycaemia, and explored additional clinical variables present in high proportion of the group of infants with no recorded BAPM hypoglycaemia risk factors.

Supplementary Table 4: Interrupted Time Series Model – Term infants

	**Coefficient (β)**	**95% CI**	**p-value**
Intercept (Baseline rate)	4.68	(4.20, 5.10)	<0.001
Pre-intervention trend	0.0025	(-0.009, -0.01415)	0.673
Intervention (Step-change)	-0.8938	(-1.46,-0.32)	0.0029
Post-intervention trend	-0.0344	(-0.05, -0.01)	0.0065

Supplementary Table 5: Interrupted Time Series Model – Term infants model Diagnostics

**Statistic**	**Value**
Durbin-Watson	1.91
Log-likelihood	54.41357 (df=6)
Pseudo - R-squared	0.6262
Residual Standard Error	0.4410423

Supplementary Table 6: Risk factors for hypoglycaemia and outcomes for infants admitted to neonatal units for hypoglycaemia during epoch one (April 2014-March 2017) and epoch two (May 2017-April 2020). *For rare outcomes (fewer than 5 events in any epoch), odds ratios and 95% confidence intervals were calculated using Fisher's exact test. For more common outcomes, odds ratios were calculated using logistic regression. Cell values of <10 are suppressed to protect confidentiality.*

	**Epoch 1 (April 2014-March 2017)**	**Epoch 2 (May 2017-April 2020)**	**OR (95% CI)****
**Term (≥37 weeks) live births, ONS, n**	**2,084,521**	**1,959,256**	
Number of term admissions primarily for hypoglycaemia, n (%)	**10,259**	**4,617**	
**BAPM Risk factors for hypoglycaemia**			
DM/GMD, n (%)	2,717 (26%)	1,268 (27%)	
HDP, n (%)	1,320 (13%)	652 (14%)	
Growth restriction: Birthweight <2nd centile, n (%)	1,283 (13%)	479 (10%)	
No BAPM risk factor	5,434 (53%)	2,440 (53%)	
Birthweight 2nd-9.9th centile, n (%)	1,973 (19%)	886 (19%)	
Birthweight >90th centile, n (%)	1,840 (18%)	830 (18%)	
**Admission and discharge variables**			
Admission glucose [mmol/L], median [IQR]	2.0 [1.5-2.5]	1.9 [1.4-2.4]	
Admission time in minutes, median [IQR]	519 [317-880]	514 [292-872]	
Discharge time [postnatal age in days], median days [IQR]	4 [2-6]	4 [2-7]	
**Outcomes**			
Died, n (%)	<10 (<0.1%)	<10 (<0.1%)	0.75 (0.15-4.83)
Severe Brain Injury, n (%)	134 (1.3%)	62 (1%)	1.03 (0.75-1.40)
Seizure, n (%)	91 (1%)	37 (1%)	0.89 (0.59-1.33)

Supplementary Table 7: Baseline characteristics for infants admitted to neonatal units for hypoglycaemia with BAPM Risk Factors compared to those without BAPM Risk Factors

**Characteristic**	**BAPM Risk Factor* (≥1)**	**No BAPM Risk Factor***
**N**	**11,825**	**13,581**
Birth Year		
2012-2014, n (%)	4,586 (39%)	5,355 (39%)
2015-2017, n (%)	4,487 (38%)	5,188 (38%)
2018-2020, n (%)	2,752 (23%)	3,038 (22%)
Gender		
Male, n (%)	7,269 (62%)	8,857 (65%)
Female, n (%)	4,547 (38%)	4,714 (35%)
Missing, n (%)	9 (<1%)	10 (<1%)
Gestation age at delivery, median weeks (IQR)	37.5 (37.0-38.4)	37.9 (37.0-38.2)
Singleton, n (%)	11,310 (96%)	12,720 (94%)
Multiple, n (%)	516 (4.3%)	847 (6.2%)
Missing, n (%)	2 (1%)	4 (<1%)
Maternal age, median (IQR)	32 (27, 36)	31 (27, 35)
Unknown, n (%)	192 (1.6%)	363 (2.7%)
Ethnicity (maternal)		
White, n (%)	6,694 (57%)	8,178 (60%)
Asian, n (%)	1,802 (15%)	1,364 (10%)
Black, n (%)	681 (5.8%)	600 (4.4%)
Mixed, n (%)	128 (1.1%)	148 (1.1%)
Other, n (%)	220 (1.9%)	217 (1.6%)
Missing, n (%)	2,300 (19%)	3,074 (23%)
Parity		
Primiparous, n (%)	4489 (38%)	5031 (37%)

Missing, n (%)	1334 (10%)	2534 (19%))
Onset of labour		
Spontaneous, n (%)	3,566 (33%)	5,599 (48%)
Induced, n (%)	4,837 (44%)	3,904 (34%)
Not in labour, n (%)	2,527 (23%)	2,114 (18%)
Unknown, n (%)	895 (7.6%)	1,964 (14.5%)
Antenatal steroids		
Complete, n (%)	6,313 (54%)	7,384 (91%)
Incomplete, n (%)	956 (8%)	681 (8.4%)
None given, n (%)	4,513 (38%)	5,477 (40%)
Birthweight centile <2nd, n (%)	3,292 (28%)	0 (0%)
Birthweight centile between 2nd-9.9th, n (%)	1,004 (7.3%)	3,908 (29%)
Birthweight centile >90th, n (%)	2,469 (21%)	2,082 (15%)
Admission temperature 36.5-37.5°C, n (%)	8,106 (70%)	9,379 (70%)
Missing admission temperature, n (%)	200 (1.6%)	272 (2%)
Admission temp >37.5°C, n (%)	127 (1.1%)	212 (1.6%)
Admission temp <36.5 °C, n (%)	3,392 (29%)	3,718 (28%)
*Feeding during NNU admission***		
Breastfeeding, n (%)	6,559 (57%)	8,055 (61%)
Bottle feeding, n (%)	9,138 (79%)	10,016 (76%)
Cup feeding, n (%)	1,643 (14%)	1,896 (14%)
Nasogastric feeding, n (%)	8,026 (69%)	8,410 (64%)
Unknown, n (%)	242 (2%)	384 (3%)
*Milk type during NNU admission***		
Breast milk, n (%)	7,662 (65%)	9,269 (69%)
Formula milk, n (%)	11,099 (94%)	12,284 (91%)
Unknown, n (%)	64 (<1%)	108 (<1%)

[1]n (%); Median (Q1, Q3)

*BAPM Risk Factor: Maternal diabetes, Maternal hypertension, fetal growth restriction <2nd centile. **Feeding and milk type are not mutually exclusive

Supplementary Table 8: UK Neonatal Collaborative units in England and Wales

Institution	Lead
Airedale General Hospital	Dr Matthew Babirecki
Arrowe Park Hospital	Dr Anand Kamalanathan
Barnet Hospital	Dr Clare Cane
Barnsley District General Hospital	Dr Kavi Aucharaz
Basildon Hospital	Dr Aashish Gupta
Basingstoke & North Hampshire Hospital	Dr Alistair Ewing
Bassetlaw District General Hospital	Dr L M Wong
Bedford Hospital	Dr Anita Mittal
Birmingham City Hospital	Dr Lindsay Halpern
Birmingham Heartlands Hospital	Dr Pinki Surana
Birmingham Women's Hospital	Dr Matt Nash
Bradford Royal Infirmary	Dr Sam Wallis
Broomfield Hospital, Chelmsford	Dr Ahmed Hassan
Calderdale Royal Hospital	Dr Karin Schwarz
Chelsea & Westminster Hospital	Dr Shu-Ling Chuang
Chesterfield & North Derbyshire Royal Hospital	Dr Penelope Young
Colchester General Hospital	Dr Ramona Onita
Conquest Hospital	Dr Graham Whincup
Countess of Chester Hospital	Dr Joanne Dangerfield
Croydon University Hospital	Dr Jocelyn Morris
Cumberland Infirmary	Dr Yee Aung
Darent Valley Hospital	Dr Abdul Hasib
Darlington Memorial Hospital	Dr Mehdi Garbash
Derriford Hospital	Dr Alex Allwood
Diana Princess of Wales Hospital	Dr Pauline Adiotomre
Doncaster Royal Infirmary	Dr Nigel Brooke
Dorset County Hospital	Dr Abby Deketelaere
East Surrey Hospital	Dr Toria Klutse
Epsom General Hospital	Dr Sonia Spathis
Frimley Park Hospital	Sathish Krishnan
Furness General Hospital	Dr Samar Sen
George Eliot Hospital	Dr Jez Jones
Glan Clwyd Hospital	Dr Geedi Farah
Glangwili General Hospital	Dr Prem Pitchaikani
Gloucester Royal Hospital	Dr Jennifer Holman
Good Hope Hospital	Dr Pinki Surana
Great Western Hospital	Dr Stanley Zengeya
Guy's & St Thomas' Hospital	Dr Geraint Lee

Harrogate District Hospital	Dr Sobia Balal
Hereford County Hospital	Dr Cath Seagrave
Hillingdon Hospital	Dr Tristan Bate
Hinchingbrooke Hospital	Dr Hilary Dixon
Homerton Hospital	Dr Narendra Aladangady
Hull Royal infirmary	Dr Hassan Gaili
Ipswich Hospital	Dr Matthew James
James Cook University Hospital	Dr M Lal
James Paget Hospital	Dr Oluseun Tayo
Kettering General Hospital	Dr Poornima Pandey
Kings College Hospital	Dr Ravindra Bhat
King's Mill Hospital	Dr Simon Rhodes
Kingston Hospital	Dr Jonathan Filkin
Lancashire Women and Newborn Centre	Dr Savi Sivashankar
Leeds Neonatal Service	Dr Lawrence Miall
Leicester General Hospital	Dr Jonathan Cusack
Leicester Royal Infirmary	Dr Venkatesh Kairamkonda
Leighton Hospital	Dr Michael Grosdenier
Lincoln County Hospital	Dr Ajay Reddy
Lister Hospital	Dr J Kefas
Liverpool Women's Hospital	Dr Alison Bedford Russell
Luton & Dunstable Hospital	Dr Jennifer Birch
Macclesfield District General Hospital	Dr Gail Whitehead
Manor Hospital	Dr Ashok Karupaiah
Medway Maritime Hospital	Dr Ghada Ramadan
Milton Keynes General Hospital	Dr I Misra
Musgrove Park Hospital	Dr Nicola Johnson
New Cross Hospital	Dr Richard Heaver
Newham General Hospital	Dr Mohammad Alam
Nobles Hospital	Dr Prakash Thiagarajan
Norfolk & Norwich University Hospital	Dr Muthukumar
North Devon District Hospital	Dr Tiziana Fragapane
North Manchester General Hospital	Dr Ngozi Edi-Osagie
North Middlesex University Hospital	Dr Cheentan Singh
Northampton General Hospital	Dr Subodh Gupta
Northumbria Specialist Emergency Care Hospital	Jess Reynolds
Northwick Park Hospital	Dr Khadija Ben-Sasi
Nottingham City Hospital	Dr Steven Wardle
Nottingham University Hospital (QMC)	Dr Steven Wardle
Ormskirk District General Hospital	Dr Victoria Nesbitt
Oxford University Hospitals, John Radcliffe Hospital	Dr Eleri Adams
Peterborough City Hospital	Dr Katharine McDevitt
Pilgrim Hospital	Dr Ruchika Gupta

Pinderfields General Hospital (Pontefract General Infirmary)	Dr David Gibson
Poole General Hospital	Prof Minesh Khashu
Prince Charles Hospital	Dr Iyad Al-Muzaffar
Princess of Wales Hospital	Dr Kate Creese
Princess Alexandra Hospital	Dr Chinnappa Reddy
Princess Anne Hospital	Dr Mark Johnson
Princess Royal Hospital	Dr Prashanth Bhat
Princess Royal Hospital (previously Royal Shrewsbury Hospital)	Dr Patricia Cowley
Princess Royal University Hospital	Dr Rashmi Gandhi
Queen Alexandra Hospital	Dr Charlotte Groves
Queen Charlotte's Hospital	Dr Lidia Tyszcuzk
Queen Elizabeth Hospital, Gateshead	Dr Shilpa Ramesh
Queen Elizabeth Hospital, King's Lynn	Dr Glynis Rewitzky
Queen Elizabeth Hospital, Woolwich - see notes	Mrs Julia Croft
Queen Elizabeth the Queen Mother Hospital	Dr Bushra Abdul-Malik
Queen's Hospital, Burton on Trent	Dr Dominic Muogbo
Queen's Hospital, Romford	Dr Ambalika Das
Rosie Maternity Hospital, Addenbrookes	Dr Angela D'Amore
Rotherham District General Hospital	Dr Soma Sengupta
Royal Albert Edward Infirmary	Dr Christos Zipitis
Royal Berkshire Hospital	Dr Peter De Halpert
Royal Bolton Hospital	Dr Archana Mishra
Royal Cornwall Hospital	Dr Chris Warren
Royal Derby Hospital	Dr John McIntyre
Royal Devon & Exeter Hospital	Dr Nagendra Venkata
Royal Hampshire County Hospital	Dr Lucinda Winckworth
Royal Lancaster Infirmary	Dr Joanne Fedee
Royal Oldham Hospital	Dr Anitha Vayalakkad
Royal Preston Hospital	Dr Raju Narasimhan
Royal Stoke University Hospital	Dr Lee Abbott
Royal Surrey County Hospital	Dr Ben Obi
Royal Sussex County Hospital	Dr Prashanth Bhat
Royal United Hospital	Dr Stephen Jones
Royal Victoria Infirmary	Dr Richard Hearn
Russells Hall Hospital	Dr Anjali Petkar
Salisbury District Hospital	Dr Jim Baird
Scarborough General Hospital	Dr Kirsten Mack
Scunthorpe General Hospital	Dr Pauline Adiotomre
Singleton Hospital	Dr Arun Ramachandran
Southend Hospital	Dr Vineet Gupta
Southmead Hospital	Dr Faith Emery
St George's Hospital	Dr Charlotte Huddy

St Helier Hospital	Dr Salim Yasin
St Mary's Hospital, IOW	Dr Akinsola Ogundiya
St Mary's Hospital, London	Dr Lidia Tyszcuzk
St Mary's Hospital, Manchester	Dr Ngozi Edi-Osagie
St Michael's Hospital	Dr Pamela Cairns
St Peter's Hospital	Dr Vennila Ponnusamy
St Richard's Hospital	Dr Victoria Sharp
Stepping Hill Hospital	Dr Carrie Heal
Stoke Mandeville Hospital	Dr Sanjay Salgia
Sunderland Royal Hospital	Dr Imran Ahmed
Tameside General Hospital	Dr Jacqeline Birch
The Grange University Hospital	Dr Sunil Reddy
The Jessop Wing, Sheffield	Dr Porus Bastani
The Royal Free Hospital	Dr Marice Theron
The Royal London Hospital - Constance Green	Dr Divyen Shah
Torbay Hospital	Dr Siba Paul
Tunbridge Wells Hospital	Dr Se-Yeon Park
University College Hospital	Dr Giles Kendall
University Hospital Coventry	Dr Puneet Nath
University Hospital Lewisham	Mrs Julia Croft
University Hospital of North Durham	Dr Mehdi Garbash
University Hospital of North Tees	Dr Hari Kumar
University Hospital of Wales	Dr Nitin Goel
Victoria Hospital, Blackpool	Dr Chris Rawlingson
Warrington Hospital	Dr Delyth Webb
Warwick Hospital	Dr Bird
Watford General Hospital	Dr Sankara Narayanan
West Cumberland Hospital	Dr Yee Aung
West Middlesex University Hospital	Dr Elizabeth Eyre
West Suffolk Hospital	Dr Jageer Mohammed
Wexham Park Hospital	Dr Sanjay Jaisal
Whipps Cross University Hospital	Dr Caroline Sullivan
Whiston Hospital	Dr Ros Garr
Whittington Hospital	Dr Wynne Leith
William Harvey Hospital	Dr Vimal Vasu
Worcestershire Royal Hospital	Dr Anna Gregory
Worthing Hospital	Dr Katia Vamvakiti
Wrexham Maelor Hospital	Dr Brendan Harrington
Wythenshawe Hospital	Dr Ngozi Edi-Osagie
Yeovil District Hospital	Dr Megan Eaton
York District Hospital	Dr Sundeep Sandhu
Ysbyty Gwynedd	Dr Michael Cronin
